# Vegetable Essences as Antiseptic Dressings

**Published:** 1893-09

**Authors:** 


### Vegetable Essences as Antiseptic Dressings.

At a meeting of the Surgical Society, of Paris, M. Lucas-Championniere (*La Tribune Medical*) remarked that, like every one else, he had tried to find a substance capable of replacing the antiseptics now in use, which were attended by the inconvenience of being toxic and irritant. At length he had made use of the essences so well studied by M. Chamberland, who had shown that they possessed an antiseptic power next to that of sublimate. It was the essence of cinnamon which was especially made use of by M. Championniere. Applied full strength upon wounds it proved to be very irritant. The idea then occurred to dissolve it in retinol, which made a pomade of good consistence. This pomade, spread upon gauze and applied upon sutured wounds (amputation of the breast, fingers, etc.), constitutes an excellent dressing. It is not irritant, and beneath the pomade the wound remains aseptic and cicatrization goes on in a perfect manner. The other essences, those of geranium, thyme, vervain, etc., possess the same property.—*Medical Bulletin.*

